# Radical-activable charge-transfer cocrystals for solar thermoelectric generator toward information conversion

**DOI:** 10.1093/nsr/nwaf121

**Published:** 2025-03-29

**Authors:** Sheng Zhuo, Yu Dong Zhao, Yan-Xin Liu, Yun Rong, Yi-Yi Ju, Lin-Feng Gu, Si-Qi Chen, Liang Wang, Wangkai Jiang, Zuo-Shan Wang, Ying-Shi Guan, Huiting Fu, Weifan Chen, Ming-Peng Zhuo, Qingdong Zheng, Liang-Sheng Liao

**Affiliations:** State Key Laboratory of Coordination Chemistry, College of Engineering and Applied Sciences, Nanjing University, Nanjing 210023, China; School of Physics and Materials Science, Nanchang University, Nanchang 330031, China; National Engineering Laboratory for Modern Silk, College of Textile and Clothing Engineering, Soochow University, Suzhou 215123, China; Institute of Functional Nano and Soft Materials (FUNSOM), Soochow University, Suzhou 215123, China; Institute of Functional Nano and Soft Materials (FUNSOM), Soochow University, Suzhou 215123, China; School of Chemistry and Chemical Engineering, Southeast University, Nanjing 211189, China; College of Chemistry, Chemical Engineering, and Materials Science, Soochow University, Suzhou 215123, China; College of Chemistry, Chemical Engineering, and Materials Science, Soochow University, Suzhou 215123, China; National Engineering Laboratory for Modern Silk, College of Textile and Clothing Engineering, Soochow University, Suzhou 215123, China; Institute of Functional Nano and Soft Materials (FUNSOM), Soochow University, Suzhou 215123, China; National Engineering Laboratory for Modern Silk, College of Textile and Clothing Engineering, Soochow University, Suzhou 215123, China; Institute of Functional Nano and Soft Materials (FUNSOM), Soochow University, Suzhou 215123, China; College of Chemistry, Chemical Engineering, and Materials Science, Soochow University, Suzhou 215123, China; National Engineering Laboratory for Modern Silk, College of Textile and Clothing Engineering, Soochow University, Suzhou 215123, China; Institute of Functional Nano and Soft Materials (FUNSOM), Soochow University, Suzhou 215123, China; College of Chemistry, Chemical Engineering, and Materials Science, Soochow University, Suzhou 215123, China; National Engineering Laboratory for Modern Silk, College of Textile and Clothing Engineering, Soochow University, Suzhou 215123, China; College of Chemistry, Chemical Engineering, and Materials Science, Soochow University, Suzhou 215123, China; School of Chemistry and Chemical Engineering, Southeast University, Nanjing 211189, China; State Key Laboratory of Coordination Chemistry, College of Engineering and Applied Sciences, Nanjing University, Nanjing 210023, China; School of Physics and Materials Science, Nanchang University, Nanchang 330031, China; Rare Earth Research Institute, Nanchang University, Nanchang 330031, China; Jiangxi Sun-Nano Advanced Materials Technology Co., Ltd. Ganzhou 341000, China; National Engineering Laboratory for Modern Silk, College of Textile and Clothing Engineering, Soochow University, Suzhou 215123, China; State Key Laboratory of Coordination Chemistry, College of Engineering and Applied Sciences, Nanjing University, Nanjing 210023, China; Institute of Functional Nano and Soft Materials (FUNSOM), Soochow University, Suzhou 215123, China

**Keywords:** open-shell radicals, charge-transfer cocrystals, photothermal conversion, solar thermoelectric generators, information conversion

## Abstract

Solar thermoelectric generators (STEGs) that can effectively harvest solar energy and convert it into affordable electricity, provide a promising solution for self-powered wearable electronics and the Internet of Things (IoT). However, their electricity generation is often limited by the low thermal concentration or unstable temperature gradients in practical applications. Herein, we rationally designed an organic radical-activable charge-transfer (CT) cocrystal based on the open-shell radical electron acceptor of 2,6-dibromonaphthalene-1,4,5,8-tetracarboxylic dianhydride. The open-shell radical contributes to the strong near-infrared absorption and nonradiative recombination, resulting in a high photothermal conversion efficiency of 67.2% for the prepared CT cocrystal. Furthermore, the photothermal ink containing the radical-activable CT cocrystal and the transparent resin was successfully coated onto a thermoelectric generator as a cost-effective light absorber, facilely forming a high-performance STEG. Notably, the prepared STEG output a voltage of 143 mV under 1 sun irradiation, demonstrating real-time photodetection capability. We anticipate the potential applications of these cocrystals in self-powered optoelectronics, such as a non-contact and long-distance information converters.

## Teaser

A rationally designed organic charge-transfer cocrystal based on an open-shell radical acceptor demonstrates strong near-infrared absorption and high photothermal conversion efficiency of 67.2%, which is successfully applied for enhancing the electricity generation and real-time information conversion.

## INTRODUCTION

Solar thermoelectric generators (STEGs), which possess the intrinsic ability to generate electricity by directly converting solar heat, have gained significant attention in both fundamental science and practical applications [1,2], including wearable electronics [3], the Internet of Things [4], and personal thermal management [5]. Notable examples of STEGs, which integrate segmented thermoelectric legs with spectrally selective solar absorbers, have demonstrated a high open-circuit voltage of 8.9 mV at a solar irradiance of 614 W m^−2^, showcasing their great potential for powering wearable devices [6]. To date, tremendous efforts have been made to enhance electricity generation through the meticulous design of high-performance thermoelectric materials [7], optimization of device configurations [8], and regulation of the thermal dissipation between the hot and cold ends [9]. For instance, a *p*-type lead telluride with controllably generated cation defects and traps for charge carriers achieves a peak figure of merit (*zT*) value of up to 2.8 at 850 K [10]. Furthermore, a substantial and stable temperature gradient (*ΔT*) across thermoelectric materials is also crucial for dramatically increasing the electrical output [11,12]. The photothermal materials of carbon-based materials [13], metal oxides [14], polymers [15], and phase-change materials [16] present a promising opportunity to serve as portable solar absorbers for desired and cost-effective thermal concentration, thereby creating a noteworthy *ΔT* that contributes to high energy conversion efficiency [17]. Notably, a composite of polyphenol-mediated liquid metal was intentionally coated onto a thermoelectric generator, which created an impressive *ΔT* of 14.5°C and a high output voltage of 185.3 mV under 1 sun irradiation [18].

Organic charge-transfer (CT) cocrystals self-assembled from an electron donor (D) and acceptor (A), exhibit unique aggregation and outstanding physicochemical properties beyond the D/A monocomponent [19]. These cocrystals have emerged as a promising alternative in various optoelectronic applications, including organic photovoltaics [20], stimuli responsiveness [21], and room-temperature ferroelectricity [22]. Interestingly, their exceptional optoelectronic properties can be precisely engineered by carefully selecting the appropriate components to rationally adjust the intermolecular CT characteristics and packing modes [23,24]. Furthermore, their electron delocalization from the donor to the acceptor can induce a novel orbital hybridization for forming a narrow bandgap, promoting the red-shifted absorption toward notable photothermal conversion [25]. In the pioneering work by Hu *et al*., dibenzotetrathiafulvalene (DBTTF) and tetracyanobenzene (TCNB) were initially utilized to create an organic photothermal cocrystal with a photothermal conversion efficiency (PCE) of 18.8% *via* a straightforward self-assembly process [26]. Since then, tremendous efforts have been made to extend the absorption into the desirable near-infrared (NIR) region and enhance nonradiative decay to achieve a high PCE in organic CT cocrystals, addressing growing practical demands [27,28]. Typically, Wei *et al*. developed a class of host–guest CT complexes based on double-cavity cyclophane, which serve as photothermal materials for photothermal therapy, achieving a high PCE of 47.4% [29]. Open-shell radicals, characterized by their inherently half-filled bands formed by π-orbital overlap, are advantageous for engaging in CT with donors, leading to significant performance enhancements [30,31], including efficient light absorption [32]. Furthermore, these open-shell radicals can also facilitate nonradiative recombination for improved photothermal conversion [33]. Inspired by these achievements, the open-shell radicals are emerging as valuable components for the innovative design of high-efficiency organic CT photothermal cocrystals aimed at high-performance STEGs [34]. However, there are few reports on the controllable fabrication of radical-activable CT cocrystals using open-shell radicals.

Herein, we have intentionally utilized the open-shell radical of 2,6-dibromonaphthalene-1,4,5,8-tetracarboxylic dianhydride (Br_2_NDA) as an electronic acceptor for forming the organic radical photothermal CT cocrystal of coronene (COR)-Br_2_NDA (CBC), achieving a high PCE of 67.2%@ (0.367 W cm^−2^). Owing to the immense electron affinity of the radical, Br_2_NDA can easily accept electrons from COR with a high degree of electron delocalization, resulting in a narrow energy gap with a considerable absorption spectrum extending to the NIR region. Furthermore, the stable radical facilitates an ultrafast nonradiative process of excited states, endowing the prepared CBC cocrystals with magnificent light-to-heat conversion properties. Impressively, the CBC cocrystal was successfully incorporated into a transparent resin to develop a photothermal ink, which was subsequently coated onto a thermoelectric generator as a light absorber for high-performance STEGs. A high photothermal conversion temperature of 70.3°C and an output voltage of 209 mV were achieved under a simulated solar source with an intensity of 2 sun. Notably, the prepared STEG also served as a real-time information converter with non-contact and long-distance capabilities. This work provides a straightforward and universal approach to the precise design of organic radical photothermal cocrystals, offering new insights into solar-thermoelectric harvesting and information conversion.

## RESULTS

**Figure 1.**
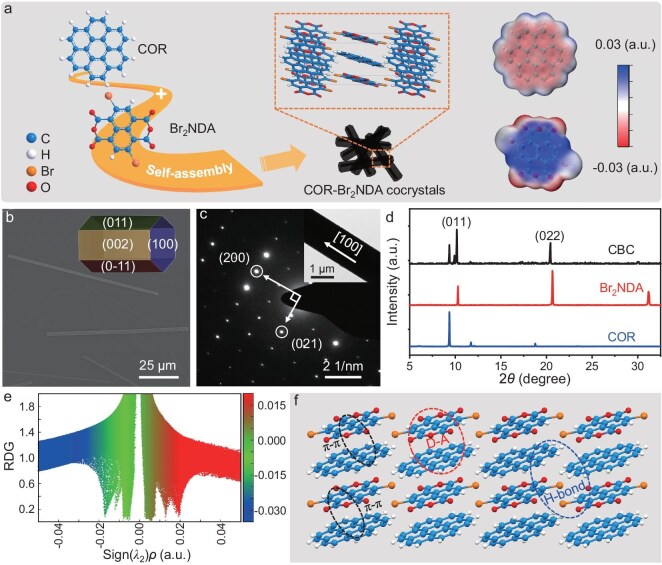
(a) Molecular structures of COR, Br_2_NDA, and CBC cocrystals, ESP distribution map, and self-assembly process. (b) SEM image of CBC cocrystals. Inset: simulated growth morphology of CBC cocrystals. (c) SAED pattern of CBC cocrystals. Inset: TEM image. (d) XRD pattern of CBC cocrystals. (e) Calculated functions of RDG and Sign (λ_2_)ρ for CBC cocrystals. (f) Crystal stacking of the CBC cocrystals.

The coronene (COR) with a well-conjugated aromatic structure features highly symmetric molecular geometry [35,36], which could controllably self-assemble into green-emissive microwires by significant π-π interactions (Fig. S1a). Meanwhile, due to the planar molecular structure and strong electron-withdrawing ability enhanced by the bromine atoms [37,38], 2,6-dibromonaphthalene-1,4,5,8-tetracarboxylic dianhydride (Br_2_NDA) is regarded in particular as an electron acceptor for designing the organic charge-transfer (CT) cocrystals. To better illustrate the point, the molecular surface electrostatic potential (ESP) distribution was calculated by density functional theory (DFT) [39] as shown in Fig. 1a. The majority of negative ESP values of the COR molecule indicate its strong electron-donating ability. In contrast, the Br_2_NDA molecule shows intense electron affinity since its ESP values were positive. Therefore, the COR and Br_2_NDA were selected as the electron donor and acceptor, respectively, to construct the organic CT cocrystals *via* a simple solution self-assembly strategy. As shown in Fig. 1a, COR and Br_2_NDA can self-assemble into needle-like CT cocrystals with metallic luster, showing no emission in contrast with the green-emissive COR microrods and the non-emissive Br_2_NDA microplates (Fig. S1b and c). The CBC cocrystal belongs to the monoclinic space group *P*2_1_/*c* with the single-crystal parameters of *a* = 7.22 Å, *b* = 9.91 Å, *c* = 17.64 Å, *α* = 90°, *β* = 90.19°, and *γ* = 90° (Table S1).

As shown in Fig. 1b, these as-prepared CBC microrods manifest a one-dimensional structure with a smooth surface, which is consistent with their simulated growth morphology (inset of Fig. 1b). Furthermore, the bright and clear diffraction spots of the selected area electron diffraction (SAED) pattern (Fig. 1c), as well as the sharp and intense characteristic diffraction peaks in the X-ray diffraction (XRD) pattern (Fig. 1d) confirm the high crystallinity of these prepared CBC microrods. The diffraction spots with measured *d*-spacing values of 3.8 and 4.8 Å at an intersection angle of 90° can be assigned to (200) and (021), respectively, which clarified the preferential growth direction of [100] for CBC microrods (Table S2). These CBC microrods exhibit various distinct diffraction peaks compared to each constituent monomer (Fig. 1d), suggesting the successful fabrication of CBC cocrystals. Moreover, the intramolecular noncovalent interactions in CBC cocrystals (Fig. 1e and Fig. S2) were studied by calculating the functions of reduced density gradient (RDG) and Sign(*λ*_2_)ρ. The larger steric hindrance (brown region) and obvious intramolecular attractive interactions (green region) in CBC cocrystals illustrate the strong CT interaction in CBC cocrystals, which is beneficial for an orderly self-assembly process along the [100] direction. Notably, donors and acceptors adopt a face-to-face pack mode, resulting in a mixed-stack structure of the CBC cocrystal as presented in Fig. 1f and Fig. S3. The donor-acceptor (D-A) distance of 3.388 Å, and donor-donor (D-D) distance of 3.609 Å suggest significant CT interaction and intermolecular π-π interaction, respectively (Fig. S4a and b), which is consistent with the above theoretical calculation. Furthermore, multiple hydrogen bonds of C─H···Br and C─H···O in CBC cocrystals contribute to the cocrystallization process (Fig. S4c). All of these results confirm the successful preparation of organic CBC cocrystals through a facile solution self-assembly process.

**Figure 2.**
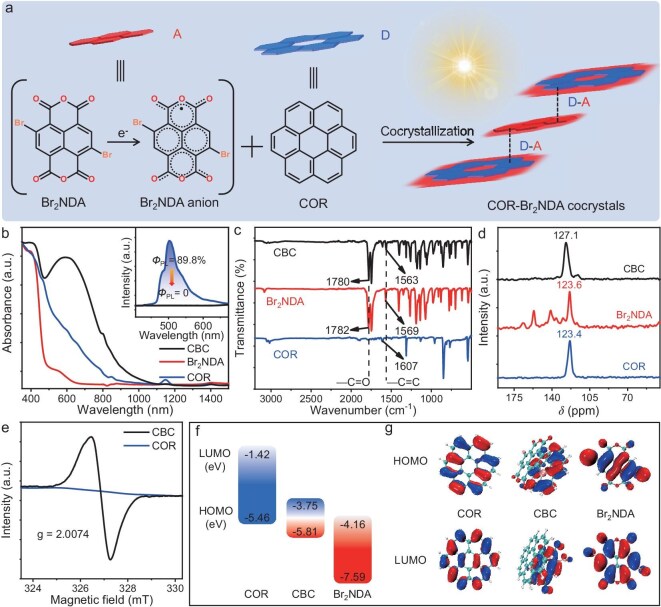
(a) Illustration of the organic CBC cocrystal formation. (b) Absorption spectra. Inset: photoluminescence spectra of COR and CBC cocrystals. (c) FTIR spectra. (d) Solid-state ^13^C NMR spectra. (e) ESR spectra. (f) Calculated energy diagrams and (g) MO diagrams of COR, Br_2_NDA, and CBC cocrystal.

Impressively, the neutral quinonoid form state of Br_2_NDA will transform into the aromatic form state of Br_2_NDA**·^−^** with active open-shell radicals and unpaired electrons after accepting an electron (Fig. 2a). Furthermore, the Br_2_NDA**·^−^** holds a strong electron affinity, which contributes to forming a strong CT interaction with COR toward the desired cocrystallization. Notably, the CBC cocrystals exhibit a broad absorption spectrum spanning 350–1100 nm (Fig. 2b). In comparison to the absorption profiles of isolated COR or Br_2_NDA, the pronounced spectral redshift directly confirms a robust charge transfer (CT) interaction between these two components [40]. Compared with the strong emission of COR at 520 nm with a high photoluminescence quantum yield (PLQY) of 89.8% (inset of Fig. 2b and Table S3), these CBC cocrystals had no emission with PLQY value close to 0, suggesting the forbidden electronic transitions of CT state and enhanced nonradiative transitions in CBC cocrystals [41]. To further verify the CT interaction between COR and Br_2_NDA, various spectroscopic tests were performed. The Fourier transform infrared (FTIR) spectra depicted in Fig. 2c show that the characteristic peaks at 1607 cm^−1^ of COR and 1569 cm^−1^ of Br_2_NDA owing to the C═C stretching vibrations are shifted to 1563 cm^−1^ in CBC cocrystals. This confirms that the electron cloud density of the benzene ring could be strengthened by the cocrystal strategy [26,42]. Furthermore, the characteristic peak at 1782 cm^−1^ of Br_2_NDA ascribed to C═O was shifted to 1780 cm^−1^ in CBC cocrystals, suggesting the charge transfer process from the donor to the acceptor. As illustrated in the solid-state ^13^C nuclear magnetic resonance (NMR) spectroscopy (Fig. 2d and Fig. S5), the chemical shift of COR at 123.4 parts per million (ppm) shows obvious downfield shifts compared with the CBC cocrystal, confirming the decline of electron density and the novel π electron delocalization from COR to Br_2_NDA. These prepared CBC cocrystals present a strong signal with a *g* factor of 2.0074 which is close to the value of a free electron (2.0032) at room temperature in the electron spin resonance (ESR) spectrum (Fig. 2e), which is absent in both COR and Br_2_NDA (Fig. 2e and Fig. S6). It suggests the existence of unpaired electrons due to the charge transfer from COR to Br_2_NDA, and the CT interaction in the ground state [25,42]. All of these results strongly support the substantial CT process from COR to Br_2_NDA molecules. Thermogravimetric (TG) and derivative thermogravimetry (DTG) analyses showed that the CBC cocrystals had good stability (Fig. S7). As shown in Fig. 2f, the highest occupied molecular orbital (HOMO) of the CBC cocrystal (−5.81 eV) is close to that of COR (−5.46 eV), and the lowest unoccupied molecular orbital (LUMO) of the CBC cocrystal (−3.75 eV) approaches that of Br_2_NDA (−4.16 eV). It clarifies that the CT process from the HOMO of COR to the LUMO of Br_2_NDA leads the rearranged electron cloud to form new molecular orbitals (MOs). From the MO diagrams of the CBC cocrystal (Fig. 2g), its HOMO resides primarily on the electron donor of COR, while the LUMO mainly resides on the electron acceptor of Br_2_NDA, resulting in a narrow energy gap and a redshift in absorption as shown in Fig. 2b [43].

**Figure 3.**
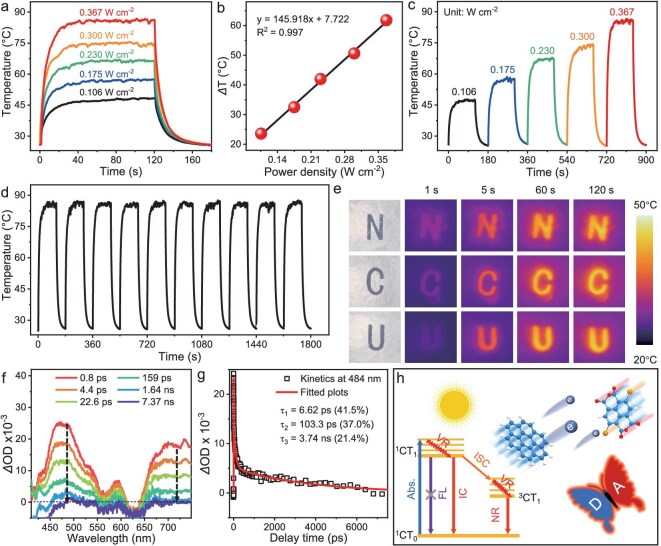
(a and c) Temperature changes at different 808 nm laser power densities. (b) Linear relationship between *ΔT* and laser power density. (d) Ten photothermal cyclic tests of CBC cocrystals at a power density of 0.367 W cm^−2^. (e) Thermal patterns of CBC cocrystals under 1 sun simulated sunlight intensity. (f) Fs-TA spectra. (g) Kinetic fitting results. (h) Jablonski diagram.

Benefiting from the intense NIR absorption, these as-prepared CBC cocrystals manifest enticing photothermal conversion performance. Under 808 nm laser irradiation with a power density of 0.367 W cm^−2^, the temperature of the CBC cocrystal powder increased sharply and reached equilibrium at 86°C within 20 s (Fig. 3a). After decreasing the power density from 0.367 to 0.106 W cm^−2^, the corresponding maximum equilibrium temperature also decreased from 86 to 46°C, revealing the excellent photothermal performance of the CBC cocrystals and fast photothermal response to NIR light. Moreover, the temperature gradient (*ΔT*) of the CBC cocrystals demonstrates a positive correlation with the power density of laser irradiation (Fig. 3b), exhibiting power density-dependent photothermal performance. Therefore, the photothermal performance of the CBC cocrystals can be adjusted in order to meet different photothermal demands by tuning the power density. As shown in Fig. 3c, the corresponding maximum equilibrium temperature is proportional to the laser power intensity. According to its linear relationship between the cooling curve and In(*θ*) as shown in Fig. S8, the photothermal conversion efficiency (PCE) of the CBC cocrystal was calculated to be 67.2% by using a previously reported method [44], which is much higher than that of reported organic photothermal CT materials as depicted in Table S4. Notably, cyclic stability was carried out as an index for the evaluation of photothermal performance. After being irradiated by an 808 nm laser with 0.367 W cm^−2^ for 10 on/off irradiation cyclic tests, the maximum equilibrium temperature of CBC cocrystals was maintained at ∼86°C (Fig. 3d and Fig. S9), which fully indicates the superior photothermal stability of the CBC cocrystals. Notably, the IR images of CBC cocrystals became brighter during the incremental increase of the irradiation time and power density (Fig. S10), fully revealing their superior photothermal imaging ability. Interestingly, the temperature of CBC cocrystals increases briskly within 30 s and stabilizes at ∼64°C under 2 sun simulated solar irradiation (Fig. S11a). After turning off the simulated solar source, the temperature gradually drops to room temperature. Likewise, under the simulated sunlight intensity of 1.5 sun, 1 sun, and 0.5 sun, the maximum equilibrium temperatures of CBC cocrystals are 55, 46, and 34°C, respectively. At the same time, the *ΔT* of CBC cocrystals is also linearly related to the simulated sunlight intensity (Fig. S11b). As shown in Fig. S12, the corresponding maximum equilibrium temperature of the CBC cocrystals maintained the same level of rise and size, indicating that these as-prepared cocrystals could be applicable for multiple cycles of heating and cooling processes with superb photothermal stability. The capital letter patterns of NCU were fabricated with CBC cocrystals to demonstrate its photothermal imaging property (Fig. 3e). The ‘N’, ‘C’, and ‘U’ patterns became brighter with the incremental increase of solar irradiation time, indicating the outstanding photothermal imaging capability of CBC cocrystals.

Femtosecond transient absorption (Fs-TA) measurements were conducted to illustrate the excited state dynamics and photothermal conversion mechanism of CBC cocrystals. After 808 nm laser excitation, a broad absorption band centered at 480 and 703 nm appears with a delay time of 0.2 ps which can be attributed to the excitation of the CT state [45], and its intensity gradually increases from 0.2 to 0.8 ps (Fig. S13). As the delay time further increases from 0.8 ps, the positive absorption peak gradually weakens and disappears at 7.37 ns. This is ascribed to the internal conversion (IC) process from CT_n_ to CT_1_, the dissociation to the charge-separated (CS) state, and the nonradiative leaps such as intersystem crossing (ISC) to the spin-triple CT state that rapidly deactivates the excited CT state (Fig. 3f) [35,46]. Furthermore, the triple exponential function of the time constants *τ*_1_, *τ*_2_, and *τ*_3_ provides a good fit to the dynamics at 484 nm (Fig. 3g). The corresponding first decay lifetime *τ*_1_ of 6.62 ps (41.5%) reveals the IC process from the excited state ^1^CT_1_ to the ground state ^1^CT_0_. The main IC process accelerates the excited state molecules to realize the energy dissipation cycle. The second lifetime *τ*_2_ is 103.3 ps (37.0%), indicating charge transfer to the CS state, and then back to the ground state *via* the IC process. The third lifetime *τ*_3_ of 3.74 ps (21.4%) illustrates the ISC transition to the ^3^CT_3_ state, then back to the ground state through the IC process. Combined with the PLQY result of CBC cocrystals, this rapid IC process proves that the radical promotes the predominant nonradiative decay pathway [28,47]. As shown in Fig. 3h, the Jablonski diagram illustrates the photophysical process of photothermal conversion based on excited state decay kinetics. These nonradiative decay processes favor good photothermal conversion performance through fine engineering of organic CT cocrystals. Additionally, the existence of Br_2_NDA**·^−^** facilitates the substantial fluorescence quenching of the CBC cocrystals leading to a PLQY of ∼0, which promotes nonradiative transition and further boosts the PCE [32,48].

**Figure 4.**
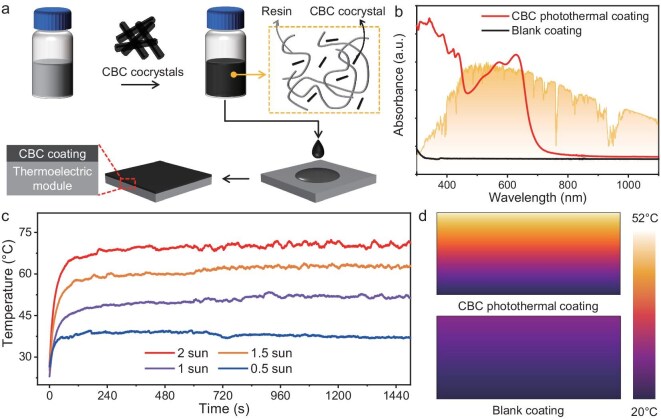
(a) Schematic diagram of the preparation of CBC photothermal coating. (b) Absorption spectra of CBC photothermal coating. (c) Temperature changes of CBC photothermal coating under irradiation by mimic solar source with different solar intensities. (d) Comparison of the photothermal effect of CBC photothermal coating and blank coating simulated by COMSOL.

Conceivably, the impressive light-harvesting capacity and superior photothermal conversion performance of CBC cocrystals could be incorporated into a thermoelectric generator (TEG), which presents a promising solution for photo-thermoelectric conversion [49]. The photothermal coatings were fabricated *via* a two-step approach as schematically depicted in Fig. 4a. First, transparent resin and CBC powders were mixed together and stirred vigorously to evenly disperse the cocrystal powders, then CBC dispersion with a deep blue color was obtained (Fig. S14). Second, the CBC dispersion was deposited onto a substrate to obtain the final photothermal coating (Fig. S15). As shown in Fig. 4b, the absorption spectrum of the CBC coating is mostly located on the visible light and NIR region compared with the blank resin, which is in agreement with the CBC cocrystals. Specifically, the CBC coating demonstrates good stability verified by TG and DTG results (Fig. S16). Under 2 sun irradiation of a simulated solar source, the maximum surface equilibrium temperature of the CBC coating reached 70.3°C within 120 s and maintained for over 1500 s. Furthermore, the corresponding maximum equilibrium temperature declined from 70.3 to 38.7°C with a decrease in solar intensity from 2 to 0.5 sun. These results highly suggest that the CBC coating holds excellent photothermal properties and long-term photothermal stability. The IR image of CBC coating under 1 sun irradiation was bright and uniformly distributed (Fig. S17), indicating the CBC cocrystal was evenly dispersed in the resin leading to the superb solar photothermal homogeneity of the CBC coating. In particular, the photothermal effect of the CBC coating was simulated *via* the light-harvesting module of COMSOL to further verify the reliability of the experimental data (Fig. S18). As shown in Fig. 4b, most of the solar irradiation could be harvested by the CBC coating, which facilitates an improved photothermal concentration of the surface area in comparison with the blank coating. The simulated maximum photothermal conversion temperature of the CBC coating was reached at ∼52°C, which was in accordance with the experimental data.

**Figure 5.**
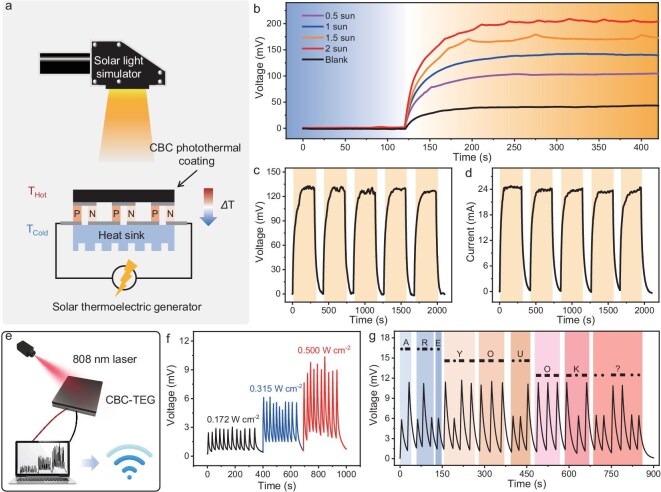
(a) Schematic diagram of CBC-TEG. (b) The output voltage of CBC-TEG under mimic solar source with different solar intensities. Cyclic test of CBC-TEG under 1 sun solar irradiation. (c) Output voltage and (d) current. (e) Schematic diagram of the information transmission by CBC-TEG. (f) The change of output voltage of CBC-TEG under different 808 nm laser power intensities. (g) The signal waveform of the Morse Code of ‘ARE YOU OK?’ by the CBC-TEG.

Following the design concept as mentioned above, the TEG module (TEG 1–127–3.6–3) was integrated with the CBC photothermal coating for novel solar thermoelectric generation as illustrated in Fig. 5a. There is a great distinction in voltage output between devices with or without a CBC photothermal coating as shown in Fig. 5b. Notably, the solar thermoelectric generation presents an output voltage of 209 mV under 2 sun irradiation, which is 375% as large as that for the bare TEG device. Furthermore, the CBC-TEG device demonstrated an enhanced voltage generation of 179, 143, and 105 mV upon solar intensities of 1.5, 1.0, and 0.5 sun, respectively. Hence, the TEG coupling with CBC photothermal coating could effectively extend the working temperature range (*ΔT_max_*), which introduces improvements in thermoelectric generation. The corresponding stability of CBC-TEG under solar light with different irradiation intensities was also investigated. After 5 heating and cooling cyclic tests under a simulated solar source with a power density of 1 sun, the maximum output voltage and current could be consistently maintained at ∼130 mV and ∼25 mA, respectively (Fig. 5c and d). Moreover, the maximum output voltage of CBC-TEG was retained at ∼98, ∼160, and ∼200 mV, respectively, after cyclic tests under 0.5, 1.5, and 2 sun of solar intensity (Fig. S19). A similar maximum output current could be achieved under different solar intensities (Fig. S20). All of these results confirmed the outstanding reusability of light-harvesting and excellent energy conversion from thermal power to electricity.

The integration of a photothermal coating also endows the CBC-TEG with a perfect response to NIR light, which could be further employed as a non-contact information conversion device (Fig. 5e). At a fixed laser irradiation time (5 s), through an incremental increase in the power intensity of the laser from 0.172 to 0.500 W cm^−2^, the time-dependent voltage of the CBC-TEG shows a gradual increase from 3 to 9 mV (Fig. 5f). In addition, the voltage changes demonstrated a reproducible response to different power intensities. By this means, different signals could be sent through the changes in power intensities. Besides, the voltage signal fluctuations could also be provided by changing light application time at a fixed power intensity. For example, the peak height of the voltage signal was defined as a ‘dot’ when the 808 nm laser with a power intensity of 0.315 W cm^−2^ irradiated the CBC-TEG for 5 s. In contrast, the ‘dash’ could be assigned to the peak height of the voltage signal when the laser intensity was adjusted to 0.500 W cm^−2^. In an actual application scenario, the information could be encrypted by remotely controlling the CBC-TEG device to generate visual electrical signals in the form of International Morse Codes (Fig. S21). Typically, the sentence ‘ARE YOU OK?’ was remotely transmitted to the detector terminal by the CBC-TEG device by constantly regulating the intensity of the laser. In this regard, the presented device proposes an accessible method for non-contact and long-distance information conversion, suggesting its wide potential application in wearable electronics.

## CONCLUSION

In conclusion, using a facile and effective solution self-assembly strategy, the organic CT cocrystal CBC with superior photothermal conversion performance has been synthesized by incorporating the open-shell radical Br_2_NDA molecule as an electron acceptor. Due to the strong CT interaction, the energy bandgap of the CBC cocrystal was reduced with a redshift absorption close to 1100 nm. The Fs-TA measurement results confirm that the rapid nonradiative dynamic pathways of IC, ISC, and charge dissociation to the ground state induced by the active radical facilitates effective energy conversion from solar light to thermal energy. This process contributes to the high PCE of 67.2%@808 nm for the CBC cocrystals. As a proof of concept, a composite CBC coating was fabricated, achieving a desirable photothermal conversion temperature of 70.3°C under 2 sun irradiation. By integrating the enticing photothermal concentrator with a thermoelectric generator, a scalable and effortless method was demonstrated to construct a CBC-TEG device that generates an enhanced output voltage of 209 mV under 2 sun irradiation. Owing to the optimal NIR light response, the CBC-TEG device also offers the capability for non-contact and long-distance real-time information conversion. This work introduces a novel strategy for STEGs that enhances robust solar-thermoelectric harvesting and facilitates flexible information conversion. This approach provides new insights into the development and application of innovative organic photothermal materials.

## Supplementary Material

nwaf121_Supplemental_File
