# Supplementary material for: Radical-activable charge-transfer cocrystals for solar thermoelectric generator toward information conversion
Source: Natl Sci Rev. 2025 Mar 29;12(5):nwaf121. doi: 10.1093/nsr/nwaf121 (PMC12057696; doi:10.1093/nsr/nwaf121)
Supplement: nwaf121_Supplemental_File [file nwaf121_supplemental_file.pdf]

Supporting Information

# Radical-Activable Charge-Transfer Cocrystals for Solar Thermoelectric Generator toward Information Conversion

Sheng Zhuo, Yu Dong Zhao, Yan-Xin Liu, Yun Rong, Yi-Yi Ju, Lin-Feng Gu, Si-Qi Chen, Liang Wang, Wangkai Jiang, Zuo-Shan Wang, Ying-Shi Guan, Huiting Fu, Weifan Chen,* Ming-Peng Zhuo*, Qingdong Zheng,*, Liang-Sheng Liao

**Experimental details**

**1. Materials**

All reagents were commercially available and used without further purification. Coronene (COR, >98.0%) was purchased from TCI. 2,6-dibromonaphthalene-1,4,5,8-tetracarboxylic dianhydride ($Br_2NDA$, 80%) was purchased from Zhengzhou Alfa Chemical Co., Ltd. Chlorobenzene (CB, 99.9%), tetrahydrofuran (THF, 99.9%), and cyclohexanone (99.9%) were purchased from Aladdin Scientific Corp. The transparent resin was purchased from Shenzhen Fantasy Color Changing New Materials Co., Ltd.

**2. The preparation of COR-$Br_2NDA$ (CBC) cocrystal microrods**

The CBC cocrystal microrods were attained via a simple solution self-assembly process. Typically, 0.01 mmol (3.00 mg) COR was dissolved in 3 mL CB and 0.01 mmol (4.26 mg) $Br_2NDA$ was dissolved in 3 ml THF at room temperature, respectively. The COR/CB and $Br_2NDA$/THF solution were sonicated for 15 min. Then the $Br_2NDA$/THF solution was slowly added into the COR/CB solution, and the resulting mixture was dropped on a quartz substrate. The formation of needle-like CBC cocrystal was observed after the solvents completely evaporated.

**3. The fabrication of CBC-based photothermal coating**

The prepared CBC cocrystals were ground into powder using a mortar. CBC cocrystal powder was added to the transparent resin according to the mass ratio of 1:100, and then cyclohexanone (20 wt%) was added as diluent. CBC-based photothermal ink could be obtained by magnetic stirring overnight. The prepared ink was coated on the top surface of the thermoelectric generator, and the CBC-based photothermal coating was obtained after drying.

**4. Characterizations**

The morphology and size of the organic micro/nanostructures were examined by field emission scanning electron microscopy (FESEM, Hitachi, SU8100, Japan) dropping on an indium tin oxide (ITO) coated glass. The TEM images were obtained

by a transmission electron microscopy (TEM, FEI company, Tecnai G2 F20, United States). The solution was dropped on a carbon-coated copper grid, and evaporated. TEM measurement was conducted at room temperature at an accelerating voltage of 100 kV. The X-ray diffraction (XRD) patterns were measured by a D/max 2400 X-ray diffractometer with Cu Kα radiation (λ = 1.54050 Å) operated in the 2θ range from 5° to 50 °, by using the samples on the quartz. The bright optical images and fluorescence images were recorded using a fluorescence optical microscope (Leica, DM4000M, Germany) with a spot-enhanced charge couple device (Diagnostic Instrument, Inc.). The excitation source is a mercury lamp equipped with a band-pass filter (330-380 nm for UV-light). The samples were fabricated by placing a drop of solution onto a cleaned quartz, and then evaporated at room temperature. Micro-area photoluminescence (μ-PL) spectra were obtained by a homemade optical microscopy. To measure the PL spectra of individual microrod, the microrod was excited locally with a 375 nm laser focused down to the diffraction limit. The excitation laser was filtered with a 375 nm notch filter. The light was subsequently coupled to a grating spectrometer (Princeton Instrument, ARC-SP-2356) and recorded by a thermal-electrically cooled CCD (Princeton Instruments, PIX-256E). The electron spin resonance (ESR) experiments were carried out on a JES-X320 spectrometer. The ESR detection was conducted at a microwave frequency of 9.15 GHz and a microwave power of 1.0 mW at room temperature. The Raman spectra were analysed by using a Raman spectrometer (HORIBA HR Evolution, France). The IR spectra were performed on an IR-408 (Shimadzu, Japan). The $^{13}C$ NMR spectra were conducted on an AVANCE III 400 machine (Bruker, Germany). The absorption spectra were analysed with a UV3600 (Shimadzu, Japan). The femtosecond transient absorption (Fs-TA) measurements were performed based on a femtosecond Ti:sapphire regenerative amplified laser system (Coherent, Astrella-Tunable-F-1k) and Fs-TA spectrometer system (Ultrafast Systems, Helios Fire). The stress-strain behaviour was investigated by Instron 5967. Thermogravimetric Analysis (TA) was

conducted on TA Instruments TGA 1. The open-circuit voltage and short-circuit current were measured by a multimeter (Keithley 7510).

**5. Characterizations of photothermal conversation performance**

The CBC cocrystal microrods were irradiated by 808 nm laser with a power density of 0.300 W $cm^{-2}$ (Zhuhai TengXing Photoelectric Technology Co., Ltd. China) for 120 s. Then, the system cools naturally and the temperature change is obtained with an infrared thermal camera (Fotric 226). The calculation of photothermal conversion efficiency (PCE, $\eta$) was determined according to the previous method [1]. The details are as follow:

Based on the total energy balance for this system:

$$\sum_i m_i \, C_{p,i} \frac{dT}{dt} = Q_s - Q_{loss}$$

$m_i$ (763.8 mg) and $C_{p,i}$ (0.8 J/(g. ℃)) represent the mass and specific heat capacity of system components, respectively. $Q_s$ is the photothermal heat energy input by irradiating NIR laser to CBC cocrystals, and $Q_{loss}$ is thermal energy lost to the surroundings. When the temperature rises to maximum, the system is in balance.

$$Q_s = Q_{loss} = hS\Delta T_{max}$$

$h$ represents heat transfer coefficient, $S$ is the surface area, and $\Delta T_{max}$ is the maximum temperature change.

The PCE of CBC cocrystals is calculated from the following equation:

$$\eta = \frac{hS\Delta T_{max}}{I(1 - 10^{-A_{808}})}$$

$I$ is the power density (0.367 W/$cm^2$) and $A_{808}$ is the absorbance of the CBC cocrystals at the wavelength of 808 nm (0.395).

In this equation, only $hS$ is unknown. In order to get the $hS$, a dimensionless driving force temperature, $\theta$ is introduced as follows:

$$\theta = \frac{T - T_{surr}}{T_{max} - T_{surr}}$$

$T$ is the temperature of CBC cocrystals, $T_{max}$ is the maximum temperature (46.8 ℃), and $T_{surr}$ is the initial temperature (28.0 ℃).

$\tau_s$ is the time constant of system.

$$\tau_s = \frac{\sum_i m_i C_{p,i}}{hS}$$

$$\frac{d\theta}{dt} = \frac{1}{\tau_s}\frac{Q_s}{hS\Delta T_{max}} - \frac{\theta}{\tau_s}$$

When the laser is off, $Q_s = 0$, therefore

$$\frac{d\theta}{dt} = -\frac{\theta}{\tau_s}, t = -\tau_s ln\theta$$

So $hS$ could be calculated from the slope of cooling time versus ln($\theta$). Therefore, the PCE of CBC cocrystals is 67.2%.

## 6. Theoretical calculations

The theoretical calculations are carried out using the Gaussian 09 software packages. The geometries of the individual molecules were optimized at the B3LYP/6-31G (d,p) level. The ESP and excited state analysis were carried out by a wavefunction analysis tool Multiwfn [2].

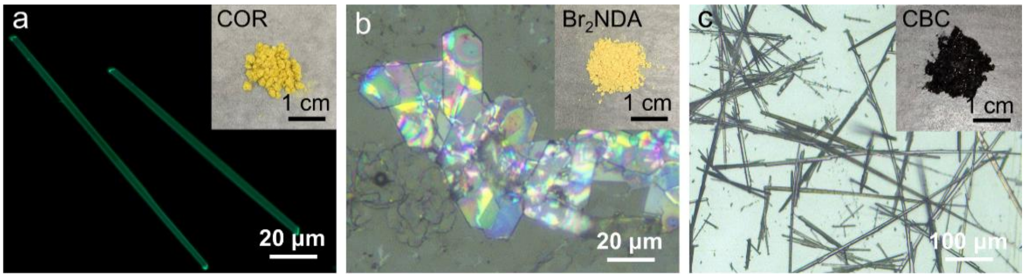


**Figure S1.** Images of (a) Coronene, (b) 2,6-Dibromonaphthalene-1,4,5,8-tetracarboxylic Dianhydride and (c) CBC cocrystals and their corresponding powders.

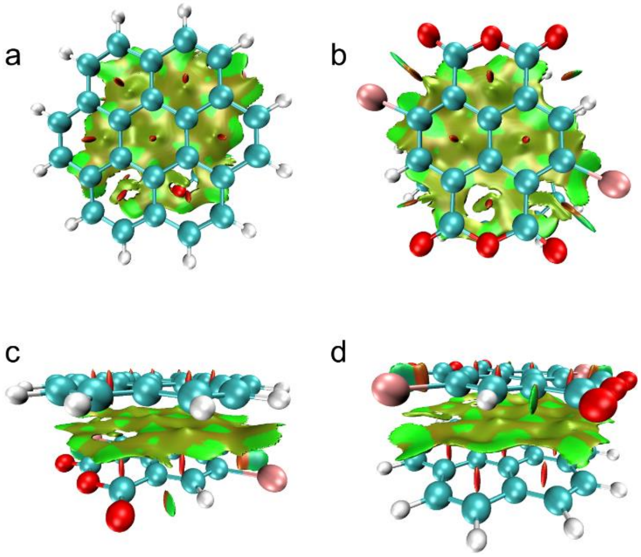


**Figure S2.** Different view of RDG isosurface map with an isovalue of 0.5 for CBC cocrystal.

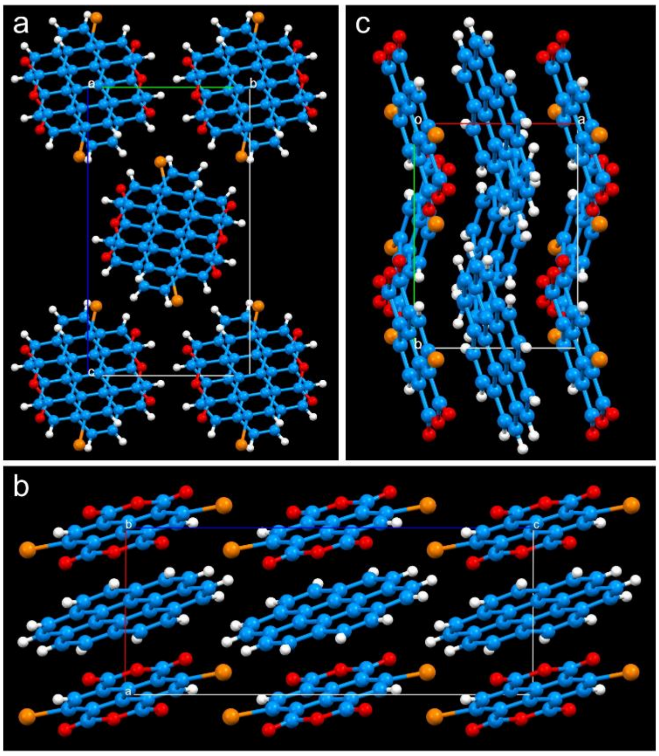


**Figure S3.** The molecular packing mode of CBC cocrystal in (a) bc, (b) ac, and (c) ab planes.

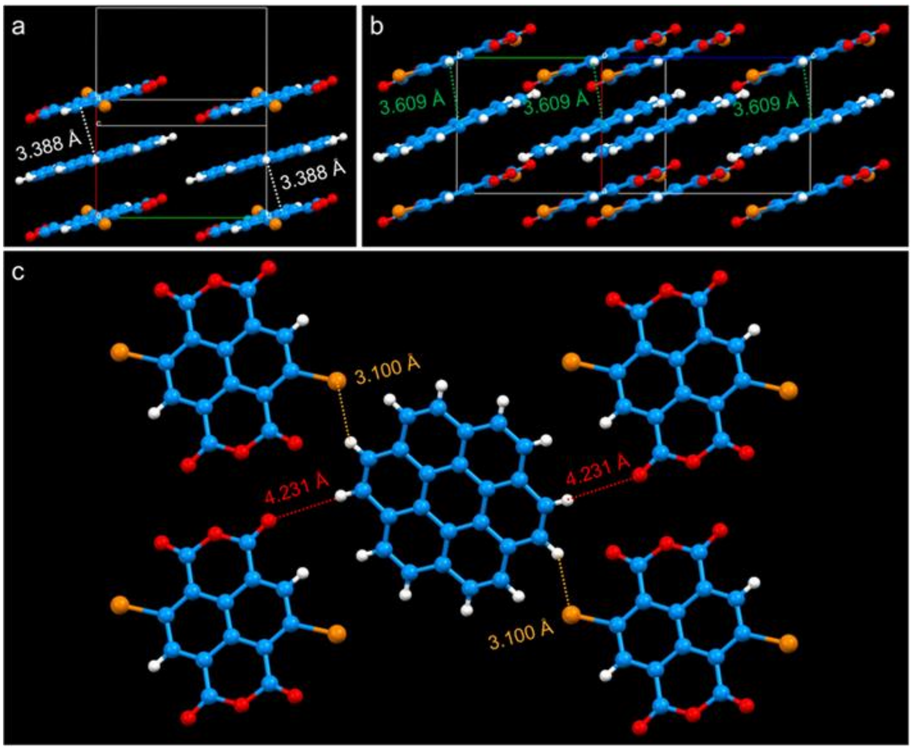


**Figure S4.** The hierarchical interactions in CBC cocrystal. (a) CT interaction, (b) π···π interaction, (c) C-H ···Br interaction, and C-H ··O interaction.

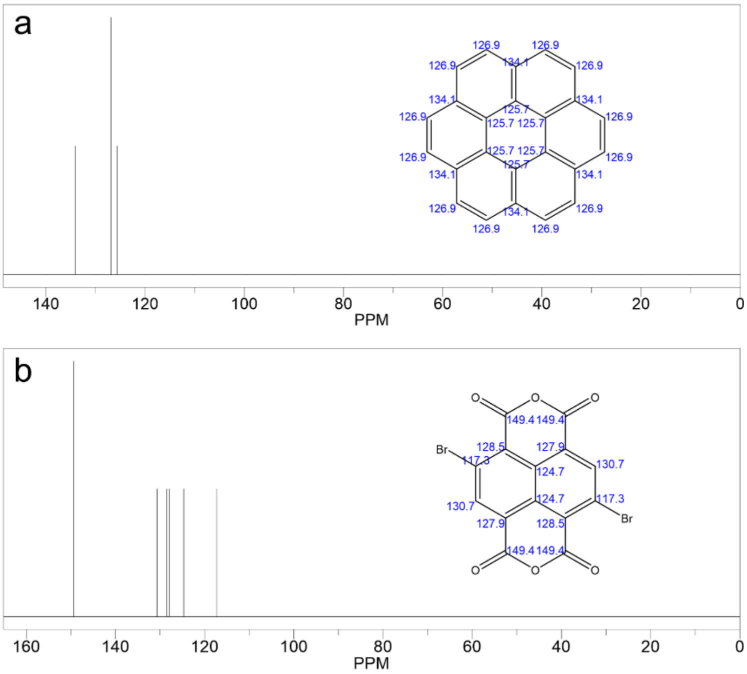


**Figure S5.** The calculated $^{13}C$ NMR spectra of (a) COR and (b) $Br_2NDA$.

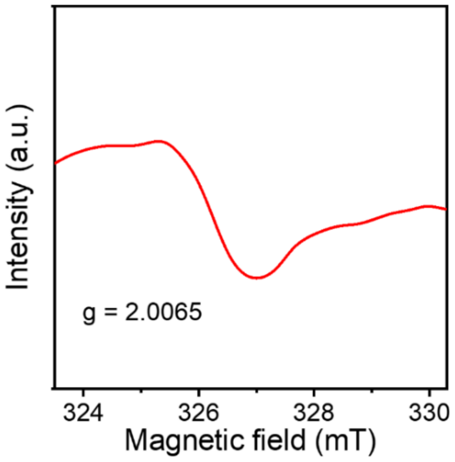


**Figure S6.** The ESR spectra of $Br_2NDA$.

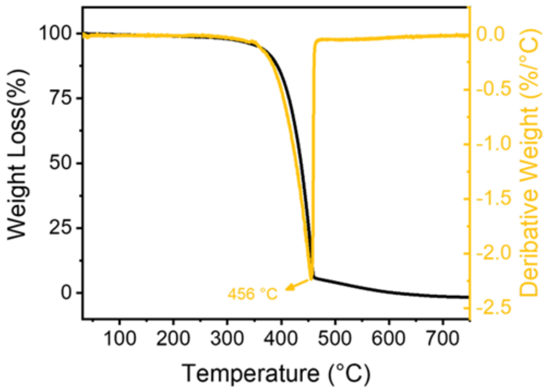


**Figure S7.** TG, and DTG results of CBC cocrystals.

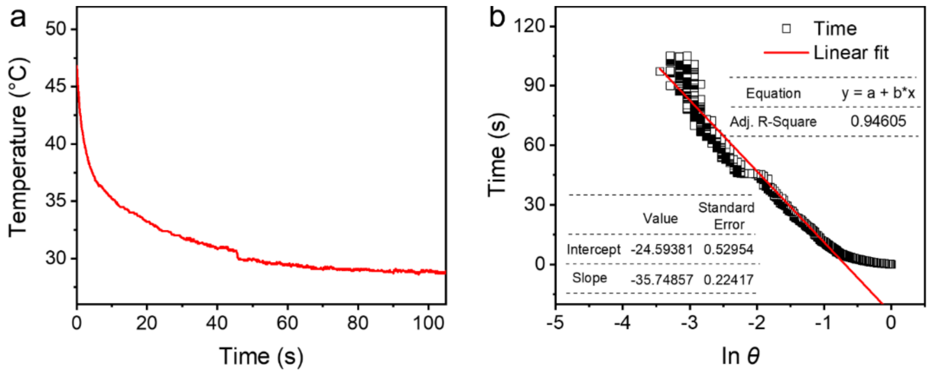


**Figure S8.** (a) The cooling curve of CDC cocrystals after irradiated by 808 nm laser with a power density of 0.367 W cm$^{-2}$. (b) The linear relationship between cooling time and In($\theta$).

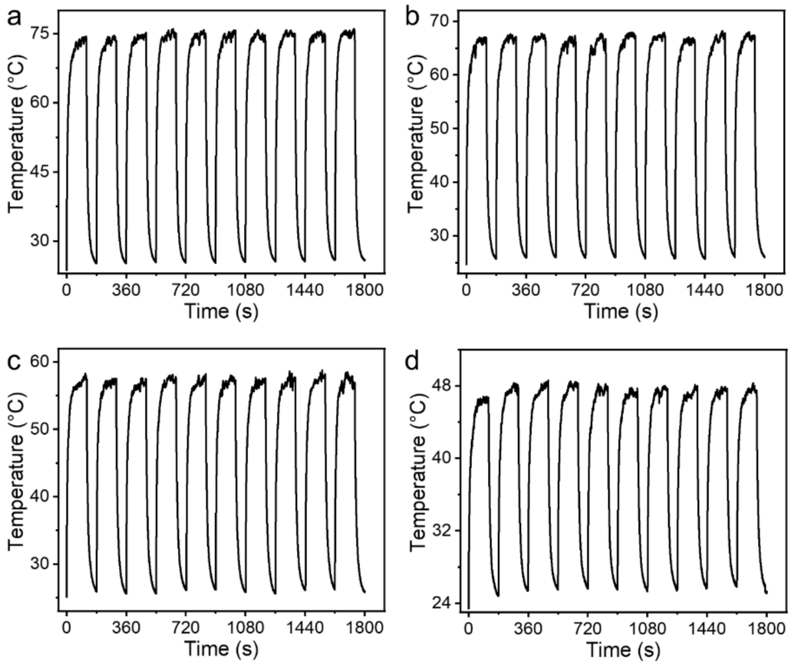


**Figure S9.** 10 photothermal cyclic tests of CBC cocrystals at different laser power densities (a) 0.300 W $cm^{-2}$, (b) 0.230 W $cm^{-2}$, (c) 0.175 W $cm^{-2}$, and (d) 0.106 W $cm^{-2}$.

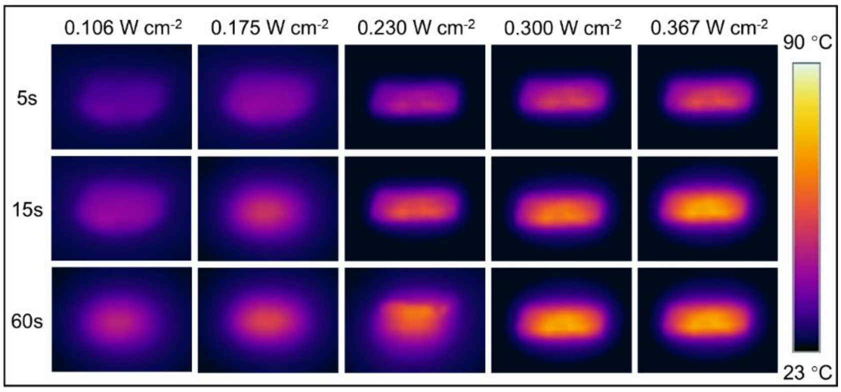


**Figure S10.** The thermal images of CBC cocrystals under irradiation by 808 nm laser with different power densities.

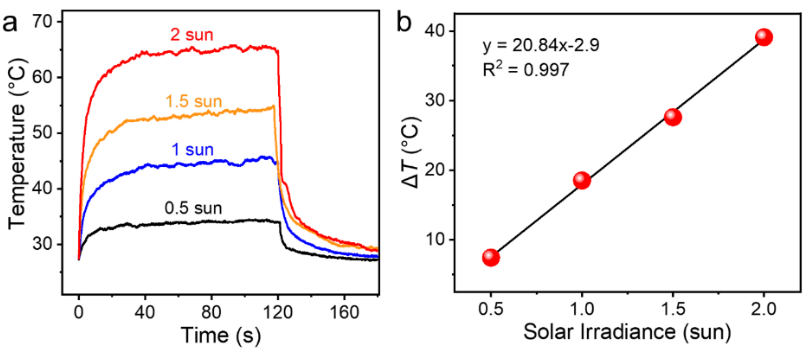


**Figure S11.** (a) The temperature changes of CBC cocrystals under different solar intensities. (b) The linear relationship between *ΔT* and solar intensities.

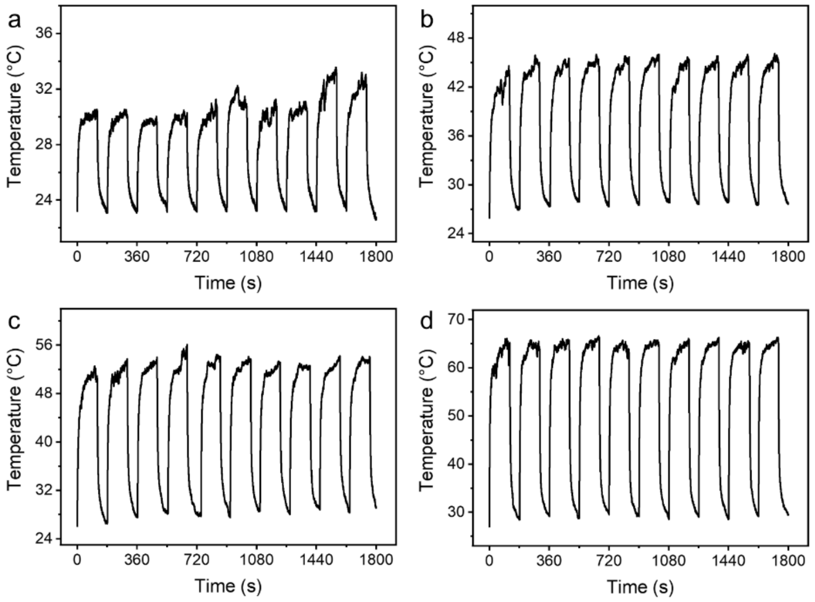


**Figure S12.** 10 photothermal cyclic test of CBC cocrystals under different solar intensities.

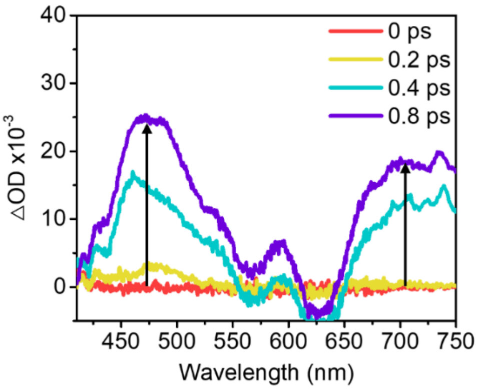


**Figure S13.** The Fs-TA spectra of CBC cocrystals at 0, 0.2, 0.4 and 0.8 ps.

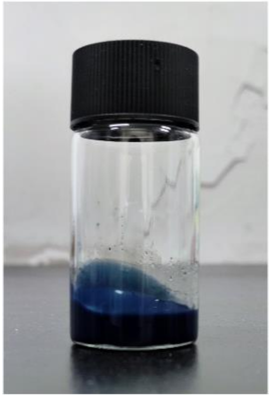

**Figure S14.** Optical image of CBC photothermal ink.

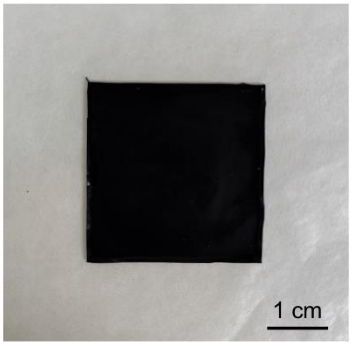


**Figure S15.** Optical image of CBC photothermal coating.

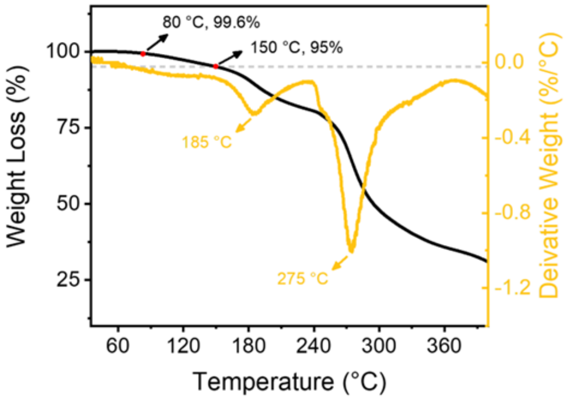


**Figure S16.** TG, and DTG results of CBC coating.

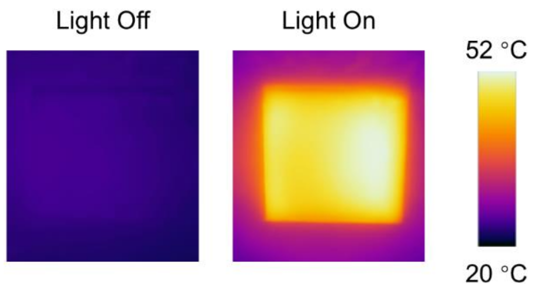


**Figure S17.** Thermal image of CBC photothermal coating under 1 sun simulated sunlight intensity.

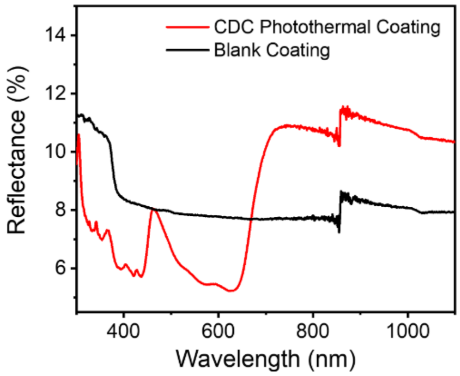


**Figure S18.** The reflectivity spectrum for COMSOL calculation.

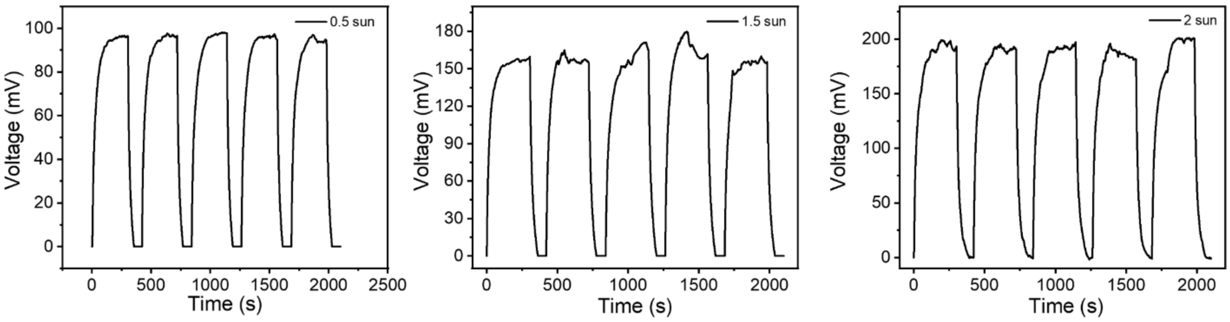


**Figure S19.** 5 photothermal cyclic (output voltage) test of CBC-TEG device under different solar intensities.

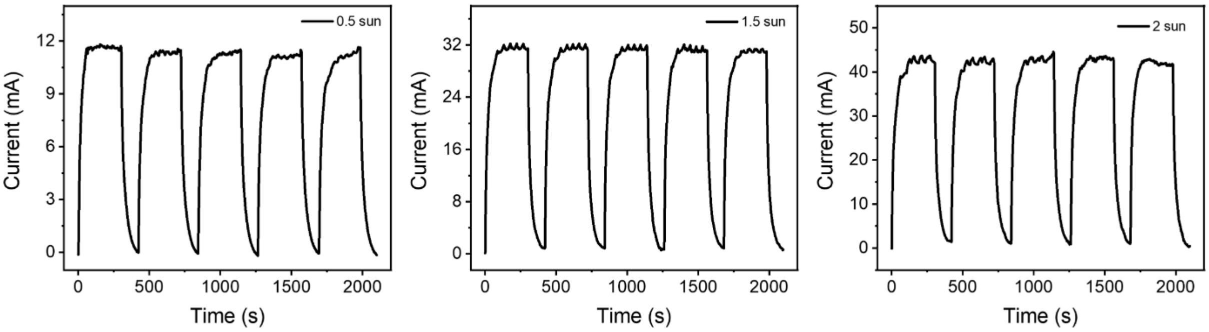


**Figure S20.** 5 photothermal cyclic test (current) of CBC-TEG device under different solar intensities.

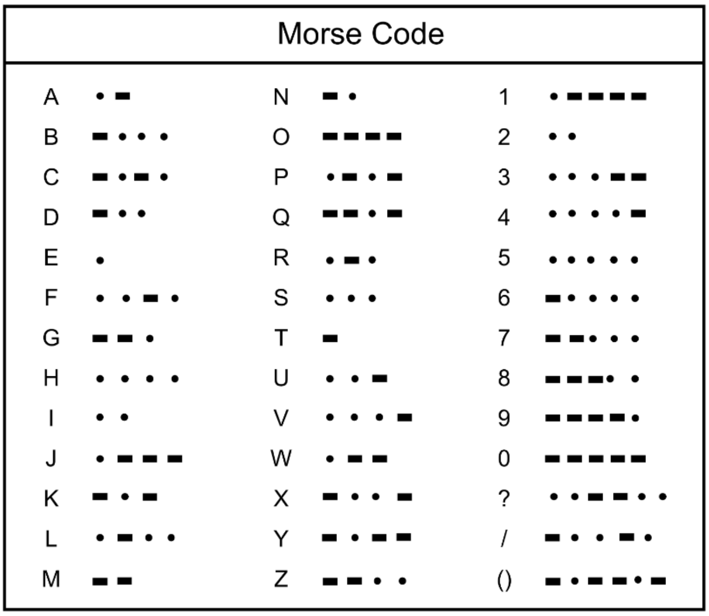


**Figure S21.** International Morse Code table.

**Table S1**. Crystal data and structure refinement for COR-$Br_2$NDA (CCDC No. 2402427).

| Name | COR-$Br_2$NDA |
|---|---|
| CCDC No. | 2402427 |
| Formula | $C_{38}H_{14}Br_2O_6$ |
| Space Group | *P 21/c* |
| | *a* = 7.2182(7) |
| Cell Lengths (Å) | *b* = 9.9125(12) |
| | *c* = 17.641(2) |
| | *α* = 90 |
| Cell Angles (°) | *β* = 90.19(5) |
| | *γ* = 90 |
| Cell Volume (Å$^3$) | 1262.2 |
| Z, Z' | Z: 2 Z': 0 |
| R-Factor (%) | 5.06 |

**Table S2**. The Attach *E*attach *{hkl}*

of various crystal facets {hkl} of COR-$Br_2$NDA cocrystal calculated by using the Material Studio software package.

| *{hkl}*s | $d_{\{hkl\}}$ (Å) | $E_{attach}$ (kcal mol$^{-1}$) |
|---|---|---|
| {002}s | 8.82 | -43.29 |
| {011}s | 8.64 | -44.62 |
| {100}s | 7.21 | -74.73 |
| {021}s | 4.77 | -55.40 |

**Table S3.** The PLQY result of COR cocrystals.

| | |
|---|---|
| Excitation Range (nm) | 300 to 400 |
| Emission Range (nm) | 400 to 800 |
| Quantum Yield (%) | 89.8 |

**Table S4.** Photothermal performance of reported charge-transfer materials.

| Materials | Laser wavelength (nm) | Absorption (nm) | PCE (%) | References |
|---|---|---|---|---|
| COR-$Br_2$NDA | 808 | 350 to 1100 | 67.2 | This work |
| Tri-PMDI-TTF | 808 | 250 to 850 | 15 | [3] |
| DBTTF-TCNB | 808 | 250 to 904 | 18.8 | [4] |
| PER-TCNQ | 1064 | 1600 | 42 | [5] |
| TMB-$F_4$TCNQ | 1064 | 1300 | 42.4 | [6] |
| TMB-TCNQ | 1064 | 1300 | 48.0 | [6] |
| GDPA-QCN | Solar light | 300-1000 | 19.33 | [7] |

## Reference


1. Kim B, Shin H, Park T, Lim H, Kim E. NIR-sensitive poly(3,4-ethylenedioxyselenophene) derivatives for transparent photo-thermo-electric converters. *Adv Mater*. 2013;25(38):5483−5489.
2. Lu T, Chen F. Multiwfn: a multifunctional wavefunction analyzer. *J Comput Chem*. 2012;33(5):580-592.
3. Wang D, Kan X, Wu C, et al. Charge transfer co-crystals based on donor-acceptor interactions for near-infrared photothermal conversion. *Chem Commun*. 2020;56(39):5223-5226.
4. Wang Y, Zhu W, Du W, et al. Cocrystals Strategy towards Materials for Near-Infrared Photothermal Conversion and Imaging. *Angew Chem Int Ed*. 2018;57(15):3963-3967.
5. Tian S, Bai H, Li S, et al. Water-Soluble Organic Nanoparticles with Programable Intermolecular Charge Transfer for NIR-II Photothermal Anti-Bacterial Therapy. *Angew Chem Int Ed*. 2021;60(21):11758-11762.
6. Ou C, Na W, Ge W, et al. Biodegradable Charge-Transfer Complexes for Glutathione Depletion Induced Ferroptosis and NIR-II Photoacoustic Imaging Guided Cancer Photothermal Therapy. *Angew Chem Int Ed*. 2021;60(15):8157-8163.
7. Liu J, Cui Y, Pan Y, et al. Donor-Acceptor Molecule Based High-Performance Photothermal Organic Material for Efficient Water Purification and Electricity Generation. *Angew Chem Int Ed*. 2022;61(14):202117087.
